# Impact of Smoking on Treatment Outcomes in Patients With Human Papillomavirus‐Related Oropharyngeal Cancer

**DOI:** 10.1002/hed.70298

**Published:** 2026-05-04

**Authors:** Michelle M. Kwan, Latifa A. Bazzi, Zequn Sun, Dayton Rand, Asher Park, Shravan Asthana, Alexis Larson, Poonam Yadav, Bharat B. Mittal, Adil Akthar, Sarah Kramer, Jochen H. Lorch, Sandeep Samant, Urjeet Patel, Michelle L. Mierzwa, Leila J. Mady, Katelyn O. Stepan, Laila A. Gharzai

**Affiliations:** ^1^ Feinberg School of Medicine Northwestern University Chicago Illinois USA; ^2^ Division of Biostatistics and Informatics, Department of Preventive Medicine Northwestern University Chicago Illinois USA; ^3^ Department of Otolaryngology Northwestern University Chicago Illinois USA; ^4^ Department of Radiation Oncology Northwestern University Chicago Illinois USA; ^5^ Division of Hematology and Oncology, Department of Medicine Northwestern University Chicago Illinois USA; ^6^ Department of Radiation Oncology University of Michigan Ann Arbor Michigan USA; ^7^ Department of Otolaryngology Johns Hopkins University Baltimore Maryland USA

**Keywords:** de‐escalation, human papillomavirus, oropharyngeal cancer, smoking history, socioeconomic status

## Abstract

**Background:**

Patients with HPV+ oropharyngeal cancer (OPC) have worse outcomes with longer smoking history (≥ 10 pack‐years). This has been confirmed in trials of primary radiotherapy but not surgery, suggesting differential smoking impact by treatment received. We assessed smoking‐related outcomes at a single institution.

**Methods:**

Kaplan–Meier and Cox proportional hazards regression compared < 10 versus ≥ 10 pack‐years by treatment received for progression‐free survival (PFS) and overall survival (OS).

**Results:**

Smoking was associated with worse PFS in both primary (chemo)radiation (HR = 1.73 [1.12–2.69], *p* = 0.01) and primary surgery cohorts (HR = 1.77 [1.02–3.07], *p* = 0.04). In surgery patients, this association was attenuated after adjusting for socioeconomic indicators (HR = 1.69 [0.97–2.93], *p* = 0.06). On recursive partitioning analysis*,* ≥ 25 pack‐years was associated with worse PFS and OS overall and among radiotherapy patients.

**Conclusions:**

Smoking impacts outcomes in HPV + OPC regardless of treatment modality. Treatment‐specific differences in prior randomized studies may reflect underlying differences in patient characteristics. ≥ 25 pack‐years was associated with worse outcomes.

## 1 Introduction

The rising incidince of human papillomavirus‐related (HPV+) oropharyngeal cancer (OPC) has reshaped risk stratification and treatment paradigms in head and neck oncology [1]. HPV + OPC has a better treatment response and a more favorable prognosis in contrast to its HPV‐unrelated counterpart, which is typically associated with older age, tobacco use, and alcohol use [2]. The landmark RTOG 0129 trial was first to demonstrate that HPV positivity is an independent prognostic factor for survival [3], and also demonstrated by recursive partitioning analysis (RPA) that patients with ≥ 10 pack‐year smoking histories had significantly worse overall survival (OS) than those with less. These criteria have since been widely used as inclusion criteria in clinical trials exploring treatment de‐intensification in this population.

RTOG 0129 was a primary chemoradiation study. In contrast, ECOG‐ACRIN 3311 was the first large national cooperative group study of primary surgically‐managed HPV + OPC [4]. After FDA approval of transoral robotic surgery (TORS) in 2009, primary surgical management of OPC steadily increased, and ECOG‐ACRIN 3311 sought to investigate differing adjuvant approaches based on pathology findings. [5, 6]. In a recent update of this study, there was no difference in progression‐free survival (PFS) or OS in patients by smoking status, using either a 10 pack‐year cut‐off (as validated in RTOG 0129) or assessing current smoking [7]. These results highlight a potential interaction between smoking history and treatment modality, suggesting that smokers may have improved outcomes with primary surgical management as compared to primary radiotherapy. However, along with limitations of cross‐trial comparisons, there are inherent differences in patients who are eligible to receive surgical management. Patients undergoing primary radiation are typically older, with more advanced disease, and a greater burden of comorbidities [8]; further, surgical resectability of the primary itself may have implications on oncologic outcomes [9].

We sought to contextualize findings from RTOG 0129 and ECOG‐ACRIN 3311 by investigating differences in outcomes for patients with HPV + OPC treated at a single institution by treatment modality and smoking status. Additionally, we evaluate optimal smoking pack‐year cutpoints with the goal of clarifying smoking's prognostic role and guiding patient selection for de‐escalation trials.

## 2 Materials and Methods

Patients in this single‐center retrospective study were identified from institutional and departmental databases. Adult patients (≥ 18 years) diagnosed with HPV + OPC from 2010 to 2024 were eligible for inclusion. All cases were (re)staged using the 8th edition of the American Joint Committee on Cancer (AJCC) staging system for HPV + OPC. Collected parameters included demographic variables (age, gender, race, ethnicity, primary language, address/zip code), smoking pack‐year history, insurance information, staging information, primary treatment received, PFS, and OS. All patients were evaluated using p16 immunohistochemistry, and p16 positivity was used as a surrogate for HPV positivity [10] as HPV in situ hybridization testing was not consistently used at this institution until halfway through the study period. Patient addresses were converted into a national area deprivation index (ADI) value from the University of Wisconsin Neighborhood Atlas, which uses a 17‐component index comprising domains of income, education, employment, and housing quality to quantify neighborhood disadvantage [11, 12]. ADI for two patients was not available on the atlas due to high group quarters population, and ADI for one patient was not available for an out‐of‐state address; these three patients were excluded from the multivariable models in this study. This study was approved by the Institutional Review Board at Northwestern University (STU00216052).

Progression‐free survival (PFS) was defined as the time from completion of definitive treatment to the first radiological evidence of recurrent malignancy or death, whichever occurred first. Overall survival (OS) was defined as the time from completion of definitive treatment to death. Living patients were censored at their most recent follow‐up date.

An a priori cutoff of 10 smoking pack‐years was used based on RTOG 0129. The association of < 10 versus ≥ 10 smoking pack‐years with PFS and OS was assessed among all patients and by primary treatment received. Time‐to‐event was estimated using the Kaplan–Meier method, with confidence intervals (CIs) based on the log–log transformation. Median PFS and OS and their estimates at 3 and 5 years with corresponding 95% confidence intervals (CIs) were reported. PFS and OS between smoking < 10 and ≥ 10 pack‐years were compared using the log‐rank test. Cox proportional hazards regression was used to evaluate the association between < 10 versus ≥ 10 smoking pack‐years with PFS and OS. Multivariable models were adjusted for sociodemographic covariates of stage, ADI, race, gender, and age.

Recursive‐partitioning analysis (RPA) was used to identify factors that were most influential for PFS and OS. Age, stage, race, ethnicity, smoking pack‐years, primary treatment, and ADI were included as prognostic factors in the RPA. The methods used for the survival analyses and Cox proportional hazards regressions performed for < 10 versus ≥ 10 smoking pack‐years were repeated for the RPA‐derived cutoff of 25 smoking pack‐years. Statistical analyses were conducted using R version 4.5.1, several packages in R Studio, and SAS version 9.4 software (SAS Institute, Cary, NC). The significance level was set to 0.05, and all reported *p*‐values and 95% CIs are two‐sided.

## 3 Results

### 3.1 Patient Characteristics

We identified 638 patients with HPV + OPC, with a median follow‐up of 4.6 years (IQR = 2.5–7) among patients still alive at time of last follow‐up (Table 1). Patients were 89% male (*n* = 565), 91% non‐Hispanic (*n* = 579), and 85% white (*n* = 543), with a median age of 61 years (IQR = 55–68). 54% (*n* = 342) of patients were never smokers, 42% (*n* = 269) were former smokers, and 4.2% (*n* = 27) were current smokers. Of the former and current smokers, 26% (*n* = 77) had < 10 pack‐years and 74% (*n* = 219) had ≥ 10 pack‐years, with a median of 20 pack‐years (IQR = 8–30). Median ADI was 31 (IQR = 16–47). Most patients were low‐risk AJCC8 Stage I‐II (92%, *n*=588). 57% (*n* = 364) patients received primary surgery, and the remaining 43% (*n* = 274) received primary radiotherapy. Consistent with national trends of increasing surgical management of HPV + OPC over time, at the start of the cohort in 2010, 18% of patients underwent primary surgery; this proportion increased steadily over time, reaching 57% in 2024.

**TABLE 1:** Patient characteristics by < 10 versus ≥ 10 smoking pack‐years.

		Smoking pack‐years	
Characteristic	Overall (*n*=638)	< 10 (*n* = 419)	≥ 10 (*n*=219)
Smoking pack‐years			
Median (IQR)	0 (0–18)	0 (0–0)	25 (16–35)
Range	0–207	0–9	10–207
Smoking status, *N* (%)			
Never	342 (54%)	342 (82%)	0 (0%)
Former	269 (42%)	77 (18%)	192 (88%)
Current	27 (4.2%)	0 (0%)	27 (12%)
Age at diagnosis (years)			
Median (IQR)	61 (55–68)	60 (54–67)	63 (56–69)
Range	30–90	30–90	41–88
Gender, *N* (%)			
Male	565 (89%)	372 (89%)	193 (88%)
Female	73 (11%)	47 (11%)	26 (12%)
Race, *N* (%)			
White	543 (85%)	352 (84%)	191 (87%)
Black or African American	46 (7.2%)	31 (7.4%)	15 (6.8%)
Other	27 (4.2%)	20 (4.8%)	7 (3.2%)
Missing/declined	22 (3.4%)	16 (3.8%)	6 (2.7%)
Ethnicity, *N* (%)			
Hispanic/Latino	30 (4.7%)	18 (4.3%)	12 (5.5%)
Not Hispanic/Latino	579 (91%)	381 (91%)	198 (90%)
Missing/declined	29 (4.5%)	20 (4.8%)	9 (4.1%)
Primary language, *N* (%)			
Not English	11 (1.7%)	7 (1.7%)	4 (1.8%)
English	627 (98%)	412 (98%)	215 (98%)
ADI National 2022			
Median (IQR)	31 (16–47)	30 (13–46)	32 (21–52)
Range	1–94	1–94	3–91
Missing	3	3	0
T Staging, *N* (%)			
T0, T1, T2, T3	599 (94%)	398 (95%)	201 (92%)
T4	39 (6.1%)	21 (5.0%)	18 (8.2%)
N Staging, *N* (%)			
N0, N1, N2	626 (98%)	411 (98%)	215 (98%)
N3	12 (1.9%)	8 (1.9%)	4 (1.8%)
Stage, *N* (%)			
Stage 0, I, II	588 (92%)	390 (93%)	198 (90%)
Stage III	50 (7.8%)	29 (6.9%)	21 (9.6%)
Treatment, *N* (%)			
Primary surgery	364 (57%)	245 (58%)	119 (54%)
Primary radiotherapy	274 (43%)	174 (42%)	100 (46%)

### 3.2 Smoking Analysis

**FIGURE 1:**
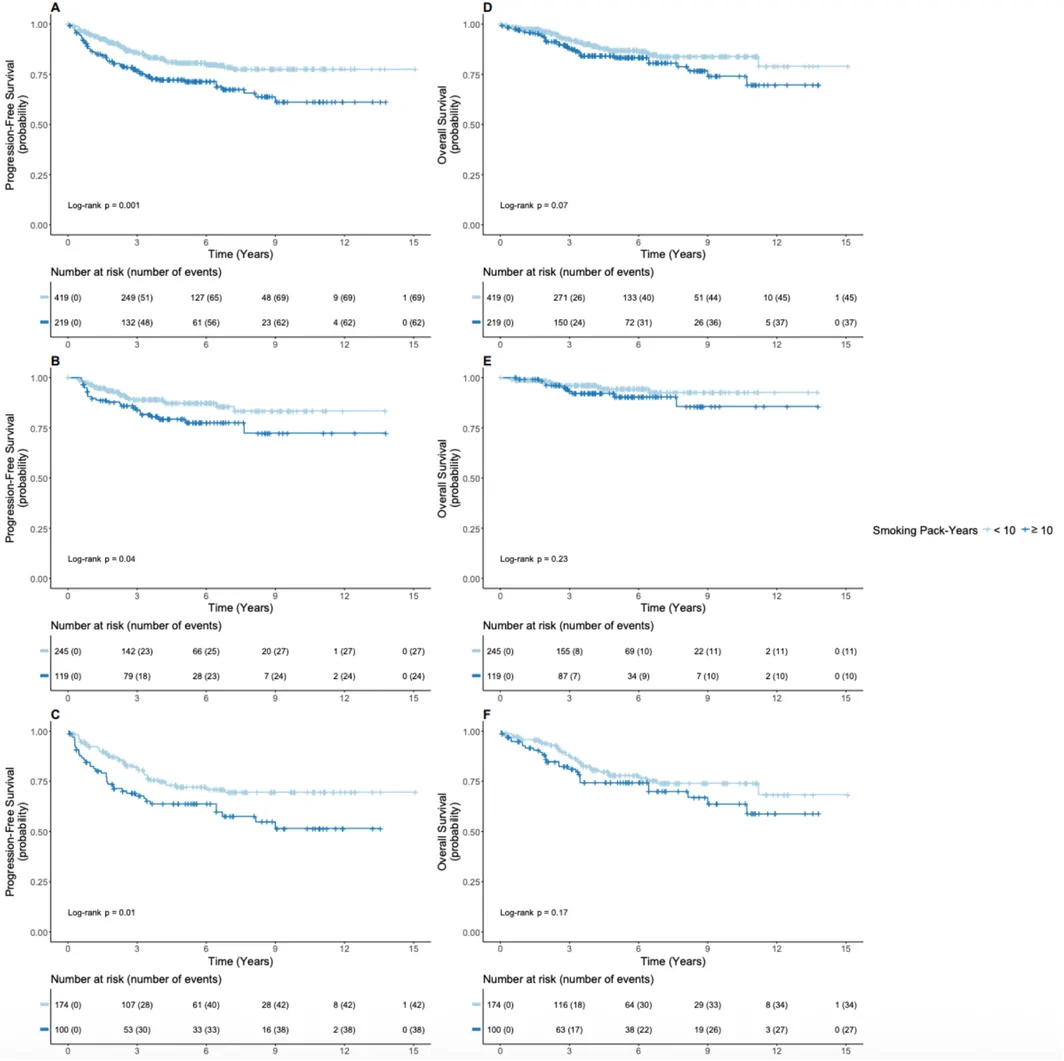
Kaplan–Meier curves for progression‐free survival and overall survival by < 10 versus ≥ 10 smoking pack‐years. Progression‐free survival for (A) overall cohort, (B) primary surgery subgroup, and (C) primary radiotherapy subgroup. Overall survival for (D) overall cohort, (E) primary surgery subgroup, and (F) primary radiation subgroup. [Color figure can be viewed at wileyonlinelibrary.com]

Median PFS was not reached (Figure 1A). The 5‐year PFS was 81% (95% CI: 76%–85%) and 72% (95% CI: 65%–78%) for < 10 pack‐years and ≥ 10 pack‐years, respectively. Smoking ≥ 10 versus < 10 pack‐years was associated with at higher risk of progression or death (HR: 1.76, 95% CI: 1.25–2.48, *p* = 0.001), which did not significantly change after adjusting for sociodemographic covariates (HR: 1.78, 95% CI: 1.26–2.51, *p* = 0.001, Table 2). Among patients who received primary surgery, 5‐year PFS was 87% (95% CI: 82%–91%) and 79% (95% CI: 70%–86%) for < 10 pack‐years and ≥ 10 pack‐years, respectively (Figure 1B). Smoking ≥ 10 pack‐years was associated with worse PFS among patients who received primary surgery (HR: 1.77, 95% CI: 1.02–3.07, *p* = 0.04), but this association was attenuated after adjusting for sociodemographic covariates (HR: 1.69, 95% CI: 0.97–2.93, *p* = 0.06). Among patients who received primary radiotherapy, 5‐year PFS was 72% (95% CI: 64%–79%) and 64% (95% CI: 53%–73%) for < 10 pack‐years and ≥ 10 pack‐years, respectively (Figure 1C). Smoking ≥ 10 pack‐years was associated with worse PFS among patients who received primary radiotherapy (HR: 1.73, 95% CI: 1.12–2.69, *p* = 0.01), which persisted after adjusting for sociodemographic covariates (HR: 1.84, 95% CI: 1.18–2.88, *p* = 0.008).

**TABLE 2:** Hazard ratios for progression‐free survival and overall survival by < 10 versus ≥ 10 smoking pack‐years.

Cohort	PFS			OS		
	*N* Events/*N* Pts	HR (95% CI) unadjusted	HR (95% CI) adjusted^a^	*N* Events/*N* Pts	HR (95% CI) unadjusted	HR (95% CI) adjusted^a^
Overall	131/638			82/638		
< 10 pack‐years	69/419	1	1	45/419	1	1
≥ 10 pack‐years	62/219	1.76 (1.25, 2.48)^b^	1.78 (1.26, 2.51)^b^	37/219	1.50 (0.97, 2.32)	1.53 (0.98, 2.39)
Primary surgery	51/364			21/364		
< 10 pack‐years	27/245	1	1	11/245	1	1
≥ 10 pack‐years	24/119	1.77 (1.02, 3.07)^b^	1.69 (0.97, 2.93)	10/119	1.69 (0.72, 3.99)	1.59 (0.68, 3.76)
Primary radiotherapy	80/274			61/274		
< 10 pack‐years	42/174	1	1	34/174	1	1
10 pack‐years	38/100	1.73 (1.12, 2.69)^b^	1.84 (1.18, 2.88)^b^	27/100	1.42 (0.86, 2.35)	1.51 (0.90, 2.54)

^a^ Multivariable models were adjusted for stage, ADI, race, gender, and age.

^b^ *p*‐value < 0.05.

Median OS was not reached (Figure 1D). The 5‐year OS was 87% (95% CI: 83%–90%) and 83% (95% CI: 77%–88%) for < 10 pack‐years and ≥ 10 pack‐years, respectively. The 5‐year OS among patients treated with primary surgery was 94% (95% CI: 90%–97%) and 90% (95% CI: 82%–95%) for < 10 pack‐years and ≥ 10 pack‐years, respectively (Figure 1E). The 5‐year OS among patients treated with primary radiotherapy was 78% (95% CI: 70%–84%) and 74% (95% CI: 63%–82%) for < 10 pack‐years and ≥ 10 pack‐years, respectively (Figure 1F). There were no differences in OS between < 10 versus ≥ 10 pack‐years in the unadjusted or adjusted models for the overall cohort (HR: 1.53, 95% CI: 0.98–2.39, *p* = 0.06) or by treatment (primary surgery HR: 1.59, 95% CI: 0.68–3.76, *p* = 0.29; primary radiotherapy HR: 1.51, 95% CI: 0.9–2.54, *p* = 0.12, Table 2).

### 3.3 Recursive Partitioning Analysis

**FIGURE 2:**
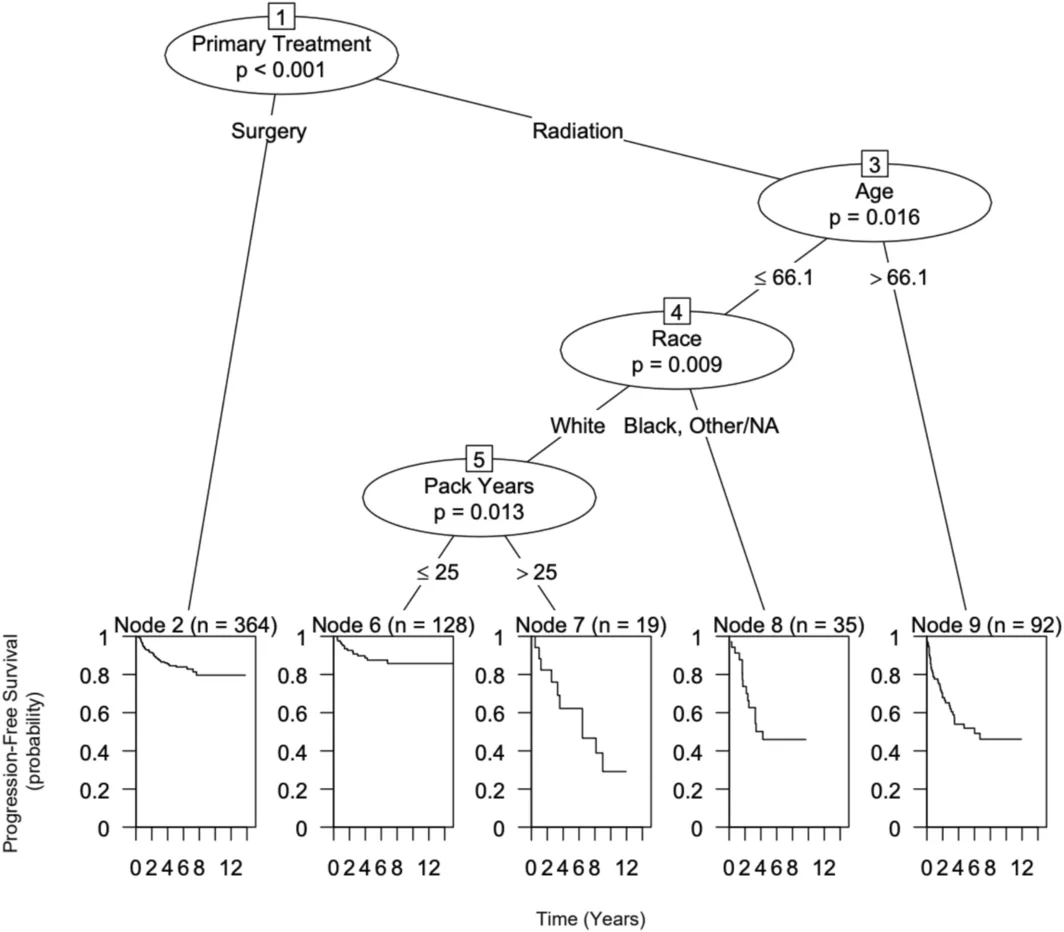
Recursive partitioning analysis for progression‐free survival.

**FIGURE 3:**
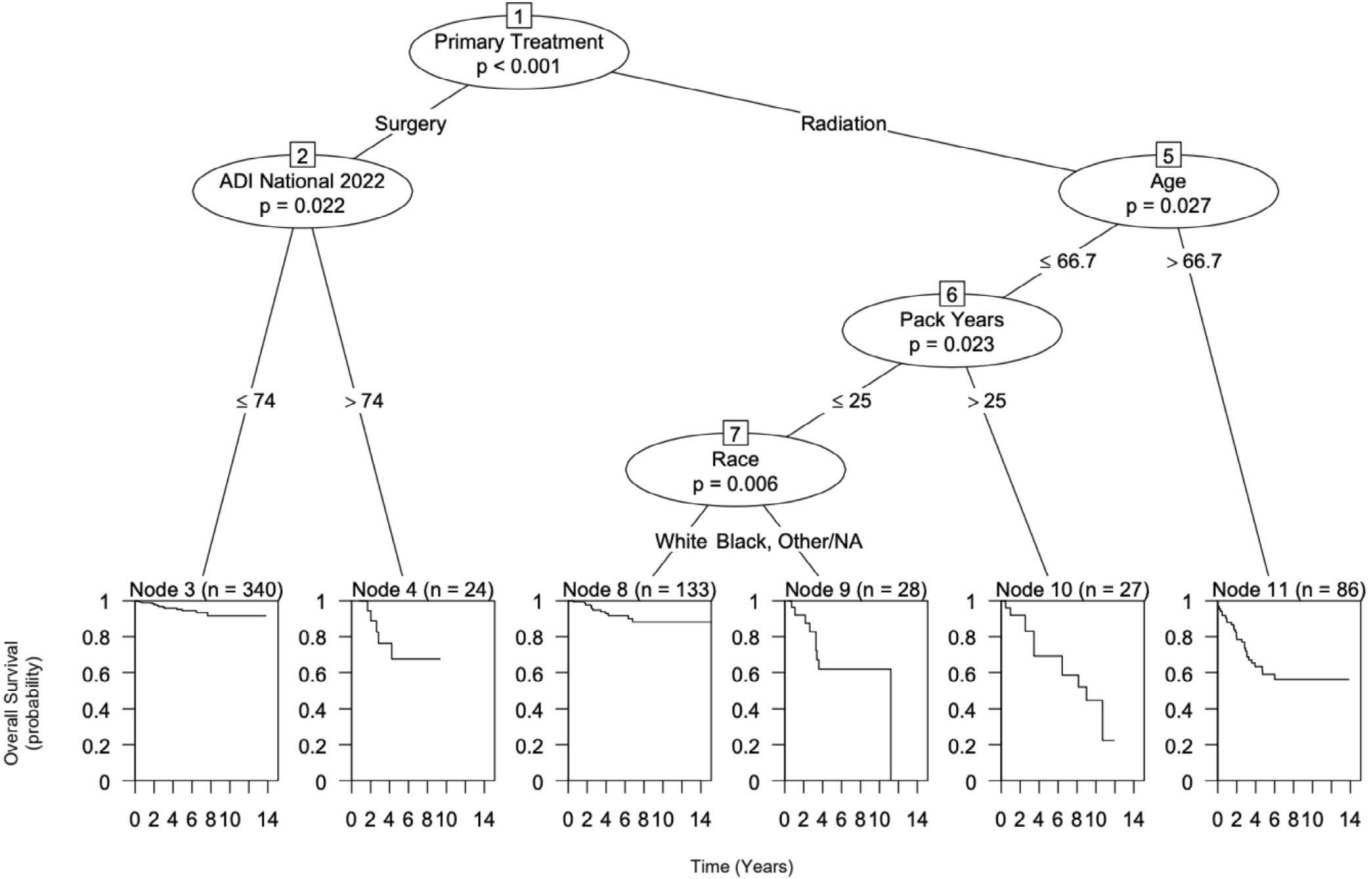
Recursive partitioning analysis for overall survival.

RPA demonstrated the most important determinant for PFS was primary treatment (surgery versus radiotherapy, Figure 2). Among patients treated with primary radiotherapy, age (≤ 66 vs. > 66) was most important, followed by race (white vs. Black/other) and smoking pack‐years (≤ 25 vs. > 25). For OS, the most important determinant was primary treatment (surgery vs. radiotherapy, Figure 3). Among patients treated with primary surgery, ADI (≤ 74 vs. > 74) further stratified OS outcomes. Age (≤ 67 vs. > 67) was most important for OS among patients treated with primary radiation, followed by smoking pack‐years (≤ 25 vs. > 25) and race (white vs. Black/other). Patient characteristics by the RPA‐derived cutpoint of 25 pack‐years are shown in Table S1.

The 5‐year PFS was 79% (95% CI: 75%–83%) and 71% (95% CI: 61%–78%) for < 25 pack‐years and ≥ 25 pack‐years, respectively (Figure S1A). Having ≥ 25 smoking pack‐years was associated with increased risk of progression or death (HR: 1.76, 95% CI: 1.20–2.59, *p* = 0.004), which remained stable after adjusting for sociodemographic covariates (HR: 1.72, 95% CI: 1.17–2.54, *p* = 0.006, Table S2). The 5‐year PFS for primary surgery was 86% (95% CI: 81%–90%) and 79% (95% CI: 65%–87%) for < 25 pack‐years and ≥ 25 pack‐years, respectively (Figure S1B). There was no association between smoking ≥ 25 versus < 25 pack‐years and PFS, even after adjusting for covariates (HR: 1.48, 95% CI: 0.77–2.86, *p* = 0.24). The 5‐year PFS for primary radiotherapy was 71% (95% CI: 64%–77%) and 61% (95% CI: 46%–73%) for < 25 pack‐years and ≥ 25 pack‐years, respectively (Figure S1C). Having ≥ 25 smoking pack‐years was associated with worse PFS among patients treated with primary radiotherapy, which remained stable after adjusting for covariates (HR: 1.88, 95% CI: 1.16–3.04, *p* = 0.01).

The 5‐year OS was 87% (95% CI: 83%–90%) and 82% (95% CI: 73%–88%) for < 25 pack‐years and ≥ 25 pack‐years, respectively (Figure S1D). The 5‐year OS for patients treated with primary surgery was 93% (95% CI: 88%–96%) and 93% (95% CI: 82%–97%) for < 25 pack‐years and ≥ 25 pack‐years, respectively (Figure S1E). The 5‐year OS for patients treated with primary radiotherapy was 78% (95% CI: 71%–84%) and 69% (95% CI: 53%–81%) for < 25 pack‐years and ≥ 25 pack‐years, respectively (Figure S1F). Smoking ≥ 25 pack‐years was associated with higher risk of death, which did not significantly change after adjusting for sociodemographic covariates both for the overall cohort (HR: 1.71, 95% CI: 1.04–2.79, *p* = 0.03) and among patients treated with primary radiotherapy (HR: 1.93, 95% CI: 1.11–3.35, *p* = 0.02) (Table S2). There was no association among patients treated with primary surgery in the unadjusted (HR: 1.08, 95% CI: 0.36–3.22, *p* = 0.89) or adjusted (HR: 1.10, 95% CI: 0.37–3.31, *p* = 0.86) models.

## 4 Discussion

In this single institution study, we demonstrate that smoking ≥ 10 pack‐years impacts outcomes for patients with HPV+ OPC, regardless of primary management with surgery or radiotherapy. The effect of smoking was attenuated for the surgical cohort when controlling for stage, demographic characteristics, and ADI as a proxy for socioeconomic status, but smoking's impact remained for patients managed with primary radiotherapy. However, using an a priori cutoff of 10 pack‐years, smoking did not impact OS overall or by treatment received.

A secondary analysis of the RTOG 0129 trial was first to demonstrate that HPV positivity in OPC is strongly associated with improved survival [3]. An RPA analysis for OS in this study identified three risk groups using HPV status, smoking history, and tumor stage [3]. Low‐risk patients were defined as those with HPV‐positive tumors, ≤ 10 pack‐year smoking history, and early T/N stage disease. Intermediate‐risk patients included HPV‐positive patients with > 10 pack‐year smoking history or advanced T/N stage, as well as HPV‐negative patients with ≤ 10 pack‐years. High‐risk patients were defined as those with HPV‐negative tumors and > 10 pack‐years. This risk stratification demonstrated significant differences in OS in patients who received primary chemoradiation; this risk stratification model was subsequently reproduced in a cohort that also included patients who received primary surgery [13]. RTOG 0129 was conducted in an era when OPC was primarily surgically managed; as the use of TORS increased over time nationally, ECOG‐ACRIN 3311 was the first cooperative group study that enrolled patients treated with primary surgery. In this trial, patients received primary transoral robotic surgery (TORS) followed by pathology‐directed adjuvant therapy, with the intermediate risk group being randomized to standard versus lower doses of adjuvant radiotherapy. This study demonstrated that smoking pack‐year history (≤ 10 vs. > 10 pack‐years) did not significantly impact 3‐year PFS or OS in any arm for patients treated with primary surgery [4, 14]. The findings of ECOG‐ACRIN 3311 support other retrospective work demonstrating no impact on OS by smoking status in a primary surgical cohort (e.g., Roden et al. [15]).

Thus, it appeared that smoking may differentially affect patients dependent on primary treatment received, with the caveat of comparing across different clinical trials. We sought to assess the same question in a single institution cohort, hypothesizing that the inherent differences in patients who are candidates for surgery versus those who are not could contribute to the discrepant results. In our primary cohort stratifying by 10 pack‐years, we found a higher smoking history significantly impacted PFS for the overall cohort and for both primary treatment modalities. However, when we adjusted for demographic and socioeconomic indicators (ADI), smoking no longer significantly influenced PFS for the primary surgical cohort. This could reflect the differences seen in RTOG 0129 versus ECOG‐ACRIN 3311, where patients who undergo surgery could also be more likely to be healthier [8] and less disadvantaged than those who undergo primary radiotherapy. In our patient cohort, the absolute PFS and OS of patients who received primary surgery was substantially higher than that of those who received primary radiotherapy regardless of smoking status, reflecting the overall treatment selection of patients who undergo primary surgery, further supported by the RPA analyses confirming the importance of primary treatment on both PFS and OS. The higher rates of comorbidities [8], inherent poorer outcomes in those who are unable to undergo resection [9], and worse overall health outcomes in smokers [16, 17] may further confound comparisons. Prior work has shown that patients residing in suburban or small urban areas, were low‐to‐middle income, or who relied on Medicaid were less likely to undergo TORS [18]. Socioeconomic status in these studies was assessed using measures such as household income, zip code‐based poverty indices, and education level. This socioeconomic selectivity may explain why large de‐intensification trials like ECOG‐ACRIN 3311 did not find strong differences in outcomes by smoking status; patients with the means to access TORS are also more likely to participate in clinical trials. Indeed, clinical trial participants tend to have higher socioeconomic status and better overall outcomes compared to nontrial participants [19, 20]. Currently, heavy smoking is used to disqualify patients for radiation de‐escalation clinical trials due to concerns that smoking is associated with worse oncologic outcomes. In light of the discrepant impact of smoking on RTOG 0129 and ECOG‐ACRIN 3311, a potential conclusion from the interpretation of these studies could be that smokers should undergo primary surgical management due to potentially improved outcomes. However, the results of this current study suggest that the differences in these studies could be related to other factors rather than smoking itself, and that perhaps smoking alone should not be used to drive treatment decisions.

Tobacco use itself is closely tied to socioeconomic disadvantage. The probability of smoking increases as the cumulative number of socioeconomic and health‐related disadvantages faced by a population rises, and disadvantaged populations account for a disproportionate share of the smoking burden [21]. Supporting this, a 2023 breast cancer study that used ADI as a measure of neighborhood‐level disadvantage found that women from more disadvantaged areas experienced shorter breast‐cancer specific survival, even after controlling for comorbidities and tumor characteristics [22]. These findings help explain how ADI confounds the relationship between smoking and PFS in our study, particularly among radiation‐treated patients, where socioeconomic status is more heterogeneous. Collectively, our results underscore that socioeconomic context plays a critical role in influencing cancer outcomes, suggesting that the observed differences by treatment modality may reflect patient selection rather than true differences in treatment efficacy. In this study, we demonstrate a correlation of worse oncologic outcomes with ADI; in other words, patients residing in more socioeconomically disadvantaged or resource‐limited communities are more likely to experience disease progression or mortality. This confirms prior literature demonstrating a well‐established pattern of worse outcomes for less privileged patients with head and neck cancers, and emphasizes the need to further study the impact of disparities on outcomes to provide patients with optimal care.

In contrast to the findings of RTOG 0129, we observed no significant differences in OS between patients with < 10 pack‐years versus ≥ 10 pack‐years overall or by treatment received. Instead, our RPA identified a 25 pack‐year threshold as a meaningful cutoff for OS (and PFS). Similar to our findings, emerging evidence from other retrospective studies has reported higher pack‐year cutoffs, in the 20–30 pack‐year range, as a threshold for impacting oncologic outcomes [23, 24, 25, 26]. This is significant due to the current use of smoking history as a cutoff for identifying low‐risk patients eligible for de‐escalation studies in HPV + OPC. Collectively, this growing body of evidence suggesting that higher smoking cutoffs are more impactful in the HPV + OPC population may mean that a subset of smokers may safely benefit from treatment de‐intensification without compromising survival outcomes. It is also important to acknowledge the health equity implications of restrictive eligibility criteria. Tobacco use is unevenly distributed across socioeconomic and racial groups, and certain populations experience lower rates of smoking cessation [27, 28]. By enforcing stringent pack‐year limits, such as those seen in current de‐escalation studies, current trials may risk inadvertently excluding patients from historically underserved backgrounds; this not only limits patient access to less toxic treatment approaches but also limits the generalizability of trial findings. Broadening smoking‐related eligibility thresholds, informed by the results of this study and other emerging data, can improve the inclusivity and external validity of future de‐escalation research.

Limitations of this study include its retrospective design. Treatment allocation was not randomized, and thus inherent differences exist between patients eligible for primary surgery versus primary radiation. Supporting this, our study demonstrated overall worse PFS and OS for primary radiotherapy patients, suggesting that imbalance existed between these cohorts. In addition, patient smoking history was self‐reported, which introduces recall bias. Furthermore, this cohort consisted of those diagnosed at a single institution with a median ADI of 31, suggesting a relatively socioeconomically advantaged cohort. This may limit the generalizability of our findings. There are also limitations associated with the use of ADI to represent socioeconomic status; ADI is an area‐level measure and may not accurately reflect individual‐level socioeconomic risk, and we utilize patients' addresses at one timepoint which may not reflect deprivation based on previous addresses.

## 5 Conclusion

In our single‐institution study, smoking history (defined as ≥ 10 pack‐years) significantly decreases PFS, regardless of primary treatment received. Among those who received primary surgery, including additional racial and sociodemographic variables attenuates smoking's impact on PFS. This suggests that some of the differences seen in randomized studies (e.g., RTOG 0129, ECOG‐ACRIN 3311) by smoking status may reflect indirect effects mediated through patient selection, comorbidities, and socioeconomic factors, rather than smoking alone; particularly if patients with better performance status or higher socioeconomic status were more likely to receive primary surgery. We also find heavy smoking (≥ 25 pack‐years) may serve as a more appropriate cutoff for evaluating smoking's impact of OS. While this study does not directly evaluate de‐escalation outcomes, our findings suggest that smoking thresholds currently used in patient stratification in ongoing treatment de‐escalation trials warrant reassessment.

## Conflicts of Interest

J.H. Lorch reports serving on advisory boards for Fore, Bayer, Eisai, and Johnson and Johnson and has received research support from Exelixis and Regeneron. The other authors declare no conflicts of interest.

## Supporting information


**Table S1:** Patient characteristics by < 25 versus ≥ 25 smoking pack‐years.
**Figure S1:** Kaplan–Meier curves for progression‐free survival and overall survival by < 25 versus ≥ 25 smoking pack‐years.
**Table S2:** Hazard ratios for progression‐free survival and overall survival by < 25 versus ≥ 25 smoking pack‐years.

## Data Availability

The data that support the findings of this study are available on request from the corresponding author. The data are not publicly available due to privacy or ethical restrictions.
