# Supplementary material for: Impact of Smoking on Treatment Outcomes in Patients With Human Papillomavirus‐Related Oropharyngeal Cancer
Source: Head Neck. 2026 May 4;48(10):2821–9. doi: 10.1002/hed.70298 (PMC13595481; doi:10.1002/hed.70298)
Supplement: Supplementary file 1 [file HED-48-2821-s001.docx]

**Supplementary Tables and Figures.**

**Title:** Impact of smoking on treatment outcomes in patients with human papillomavirus-related oropharyngeal cancer

**Authors:** Michelle M. Kwan BA^1^, Latifa Bazzi MPH^2^, Zequn Sun PhD^2^, Dayton Rand MD^3^, Asher Park BS^1^, Shravan Asthana BS^1^, Alexis Larson APRN^3^, Poonam Yadav PhD^4^, Bharat B. Mittal MD^4^, Adil Akthar MD^4^, Sarah Kramer SLP^3^, Jochen H. Lorch MD^5^, Sandeep Samant MD^3^, Urjeet Patel MD^3^, Michelle L. Mierzwa MD^6^, Leila J. Mady MD PhD MPH^7^, Katelyn O. Stepan MD^3^, Laila A. Gharzai MD LLM^4^

**Contents**

[Supplementary Table 1. Patient Characteristics by <25 vs. ≥25 Smoking Pack-Years.	2](#_Toc215692118)

[Supplementary Figure 1. Kaplan-Meier Curves for Progression-Free Survival and Overall Survival by <25 vs. ≥25 Smoking Pack-Years.	3](#_Toc215692119)

[Supplementary Table 2. Hazard Ratios for Progression-Free Survival and Overall Survival by <25 vs. ≥25 Smoking Pack-Years.	4](#_Toc215692120)

# Supplementary Table 1. Patient Characteristics by <25 vs. ≥25 Smoking Pack-Years.

|  | | **Smoking Pack-Years** | |
| --- | --- | --- | --- |
| **Characteristic** | **Overall**  N = 638 | **< 25**  N = 523 | **≥ 25**  N = 115 |
| **Smoking Pack-Years** |  |  |  |
| Median (IQR) | 0 (0-18) | 0 (0-5) | 35 (30-42) |
| Range | 0-207 | 0-24 | 25-207 |
| **Smoking Status, N (%)** |  |  |  |
| Never | 342 (54%) | 342 (65%) | 0 (0%) |
| Former | 269 (42%) | 172 (33%) | 97 (84%) |
| Current | 27 (4.2%) | 9 (1.7%) | 18 (16%) |
| **Age at Diagnosis (Years)** |  |  |  |
| Median (IQR) | 61 (55-68) | 61 (54-67) | 63 (56-69) |
| Range | 30-90 | 30-90 | 43-88 |
| **Gender, N (%)** |  |  |  |
| Male | 565 (89%) | 460 (88%) | 105 (91%) |
| Female | 73 (11%) | 63 (12%) | 10 (8.7%) |
| **Race, N (%)** |  |  |  |
| White | 543 (85%) | 441 (84%) | 102 (89%) |
| Black or African American | 46 (7.2%) | 37 (7.1%) | 9 (7.8%) |
| Other | 27 (4.2%) | 26 (5.0%) | 1 (0.9%) |
| Missing/Declined | 22 (3.4%) | 19 (3.6%) | 3 (2.6%) |
| **Ethnicity, N (%)** |  |  |  |
| Hispanic/Latino | 30 (4.7%) | 25 (4.8%) | 5 (4.3%) |
| Not Hispanic/Latino | 579 (91%) | 474 (91%) | 105 (91%) |
| Missing/Declined | 29 (4.5%) | 24 (4.6%) | 5 (4.3%) |
| **Primary Language, N (%)** |  |  |  |
| Not English | 11 (1.7%) | 9 (1.7%) | 2 (1.7%) |
| English | 627 (98%) | 514 (98%) | 113 (98%) |
| **ADI National 2022** |  |  |  |
| Median (IQR) | 31 (16-47) | 30 (15-46) | 36 (22-56) |
| Range | 1-94 | 1-94 | 4-91 |
| Missing | 3 | 3 | 0 |
| **T Staging, N (%)** |  |  |  |
| Tx, T1, T2, T3 | 599 (94%) | 494 (94%) | 105 (91%) |
| T4 | 39 (6.1%) | 29 (5.5%) | 10 (8.7%) |
| **N Staging, N (%)** |  |  |  |
| N0, N1, N2 | 626 (98%) | 514 (98%) | 112 (97%) |
| N3 | 12 (1.9%) | 9 (1.7%) | 3 (2.6%) |
| **Stage, N (%)** |  |  |  |
| Stage 0, I, II | 588 (92%) | 486 (93%) | 102 (89%) |
| Stage III | 50 (7.8%) | 37 (7.1%) | 13 (11%) |
| **Treatment, N (%)** |  |  |  |
| Primary Surgery | 364 (57%) | 303 (58%) | 61 (53%) |
| Primary Radiotherapy | 274 (43%) | 220 (42%) | 54 (47%) |

# Supplementary Figure 1. Kaplan-Meier Curves for Progression-Free Survival and Overall Survival by <25 vs. ≥25 Smoking Pack-Years.

Progression-free survival for (A) overall cohort, (B) primary surgery subgroup, and (C) primary radiotherapy subgroup. Overall survival for (D) overall cohort, (E) primary surgery subgroup, and (F) primary radiation subgroup.

# Supplementary Table 2. Hazard Ratios for Progression-Free Survival and Overall Survival by <25 vs. ≥25 Smoking Pack-Years.

|  | **PFS** | | | **OS** | | |
| --- | --- | --- | --- | --- | --- | --- |
| **Cohort** | **N Events**  **/N Pts** | **HR (95% CI)**  **Unadjusted** | **HR (95% CI)**  **Adjusted^†^** | **N Events**  **/N Pts** | **HR (95% CI)**  **Unadjusted** | **HR (95% CI)**  **Adjusted^†^** |
| **Overall** | 131/638 |  |  | 82/638 |  |  |
| < 25 | 95/523 | 1 | 1 | 59/523 | 1 | 1 |
| ≥ 25 | 36/115 | 1.76 (1.20, 2.59) ^‡^ | 1.72 (1.17, 2.54) ^‡^ | 23/115 | 1.73 (1.07, 2.80) ^‡^ | 1.71 (1.04, 2.79) ^‡^ |
| **Primary Surgery** | 51/364 |  |  | 21/364 |  |  |
| < 25 | 39/303 | 1 | 1 | 17/303 | 1 | 1 |
| ≥ 25 | 12/61 | 1.50 (0.78, 2.86) | 1.48 (0.77, 2.86) | 4/61 | 1.08 (0.36, 3.22) | 1.10 (0.37, 3.31) |
| **Primary Radiotherapy** | 80/274 |  |  | 61/274 |  |  |
| < 25 | 56/220 | 1 | 1 | 42/220 | 1 | 1 |
| ≥ 25 | 24/54 | 1.88 (1.17, 3.03) ^‡^ | 1.88 (1.16, 3.04) ^‡^ | 19/54 | 1.92 (1.11, 3.29) ^‡^ | 1.93 (1.11, 3.35) ^‡^ |

^†^Multivariable models were adjusted for stage, ADI, race, gender, and age.

^‡^p-value < 0.05.
